# Mesenchymal Stem Cells: Potential Role in the Treatment of Osteochondral Lesions of the Ankle

**DOI:** 10.1002/biot.201700070

**Published:** 2017-11-22

**Authors:** Howard C. Tribe, Josephine McEwan, Heath Taylor, Richard O. C. Oreffo, Rahul S. Tare

**Affiliations:** ^1^ Bone and Joint Research Group, Centre for Human Development, Stem Cells and Regeneration Faculty of Medicine University of Southampton Southampton SO16 6YD UK; ^2^ Foot and Ankle Orthopaedic Department Royal Bournemouth Hospital Bournemouth BH7 7DW UK; ^3^ Bioengineering Science, Mechanical Engineering Department Faculty of Engineering and the Environment University of Southampton Southampton SO17 1BJ UK

## Abstract

Given articular cartilage has a limited repair potential, untreated osteochondral lesions of the ankle can lead to debilitating symptoms and joint deterioration necessitating joint replacement. While a wide range of reparative and restorative surgical techniques have been developed to treat osteochondral lesions of the ankle, there is no consensus in the literature regarding which is the ideal treatment. Tissue engineering strategies, encompassing stem cells, somatic cells, biomaterials, and stimulatory signals (biological and mechanical), have a potentially valuable role in the treatment of osteochondral lesions. Mesenchymal stem cells (MSCs) are an attractive resource for regenerative medicine approaches, given their ability to self‐renew and differentiate into multiple stromal cell types, including chondrocytes. Although MSCs have demonstrated significant promise in in vitro and in vivo preclinical studies, their success in treating osteochondral lesions of the ankle is inconsistent, necessitating further clinical trials to validate their application. This review highlights the role of MSCs in cartilage regeneration and how the application of biomaterials and stimulatory signals can enhance chondrogenesis. The current treatments for osteochondral lesions of the ankle using regenerative medicine strategies are reviewed to provide a clinical context. The challenges for cartilage regeneration, along with potential solutions and safety concerns are also discussed.

## 1 Introduction

Hyaline articular cartilage (AC) covers the ends of articulating bones and its unique biomechanical characteristics reduce friction during bone movement by facilitating adsorption of mechanical load. This complex tissue is avascular, aneural, alymphatic, and is sparsely populated with chondrocytes that procure nutrients from the synovial fluid solely via diffusion. These biological characteristics severely limit the ability of AC to self‐repair. Due to the limited potential for self‐repair, defects in AC caused by acute trauma or as a consequence of more chronic pathologies, such as osteoarthritis (OA), osteonecrosis, and osteochondritis dissecans, may lead to gradual cartilage deterioration and loss.

The deterioration of AC has been recognized since the time of Hippocrates^1^ and the clinical symptoms of pain, swelling, and loss of function are well documented.^2^ With an increasing aging population, there is a high incidence of AC disorders, and the physical, psychological, and socio‐economic burden to patients and society is considerable.^3^ Over the years, a number of different surgical as well as non‐surgical strategies have been developed to treat the symptoms of articular joint deterioration, but, to date, none have been able to show the long‐term restoration of innate joint function that patients require. The inadequacy of current treatments has encouraged the proliferation of technologies that aim to regenerate the native cartilage tissue.

The discipline of regenerative medicine is defined as a scientific field that replaces or regenerates human cells, tissue, or organs to restore or establish normal function.^4^ Regeneration of articular cartilage refers to the restoration of the articular surface and mechanical integrity in order to improve function, reduce pain, and prevent end‐stage joint degeneration. Since their inception, cartilage regenerative strategies have focused primarily on the knee joint, but the ankle is also a suitable target. The incidence of ankle injuries has been recorded at 107 fractures per 10^5^ person‐years^5^ and up to 61% of fractures damage the articular surface of the ankle.^6^ Damage to the articular cartilage surface results in a chondral lesion that, if left untreated, can progress deeper and affect the underlying bone, contributing to the development of an osteochondral lesion. The technical challenges of treating articular cartilage lesions in the ankle joints compared to articular cartilage lesions in the knee joints include the difficulty in accessing all areas of the ankle joint, smaller size, and the lack of non‐weight bearing cartilage that can be utilized for regeneration strategies.

**Figure 1:**
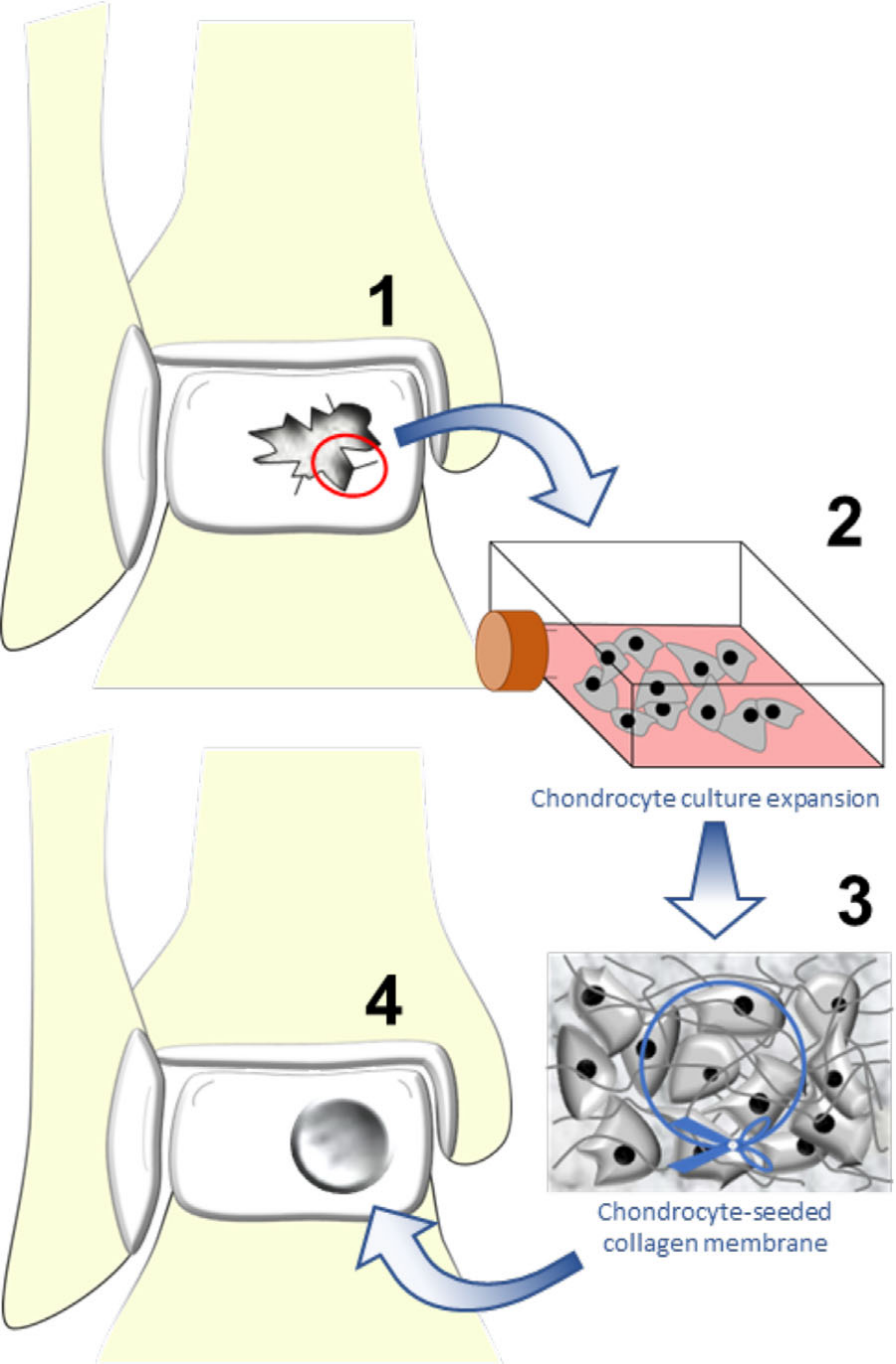
An illustration of third‐generation autologous chondrocyte implantation for the repair of a chondral lesion in the ankle. 1) During the first operation, a cartilage biopsy is taken from areas of damaged cartilage within the ankle or from the ipsilateral knee. 2) Chondrocytes are isolated from the biopsied cartilage via enzymatic digestion and cultured in 2‐D monolayer cultures. 3) Monolayer culture‐expanded chondrocytes are seeded on to a collagen types I–III membrane. 4) In the second operation, the cartilage lesion is prepared and the collagen membrane is then cut to size, placed in the lesion, and secured with fibrin glue.

Chondrocytes have been used to treat AC lesions for the last few decades using the technique of autologous chondrocyte implantation (ACI).^7^ The third generation of the technique involves taking a cartilage biopsy, culture‐expanding the chondrocytes, seeding the chondrocytes onto a collagen type‐I/III scaffold, and subsequently implanting the cellularized scaffold into the cartilage lesion (Figure 1). The technique has been shown to have favorable long‐term results when treating cartilage lesions in the ankle^8^ but a meta‐analysis in 2012 found insufficient evidence to support ACI over the simple and inexpensive technique of bone marrow stimulation.^9^ Furthermore, the two‐step procedure of ACI doubles the surgical risk to the patient, thus, chondrocytes may have to be harvested from the uninjured ipsilateral knee and the problems of culture‐expanding the cells after harvest, namely cell senescence,^10^ dedifferentiation,^11^ and cost, preclude universal adoption across health organizations.^12^ Due to the limitations associated with the use of chondrocytes, stem cells have emerged as a viable alternative for cartilage regenerative medicine strategies.

**Figure 2:**
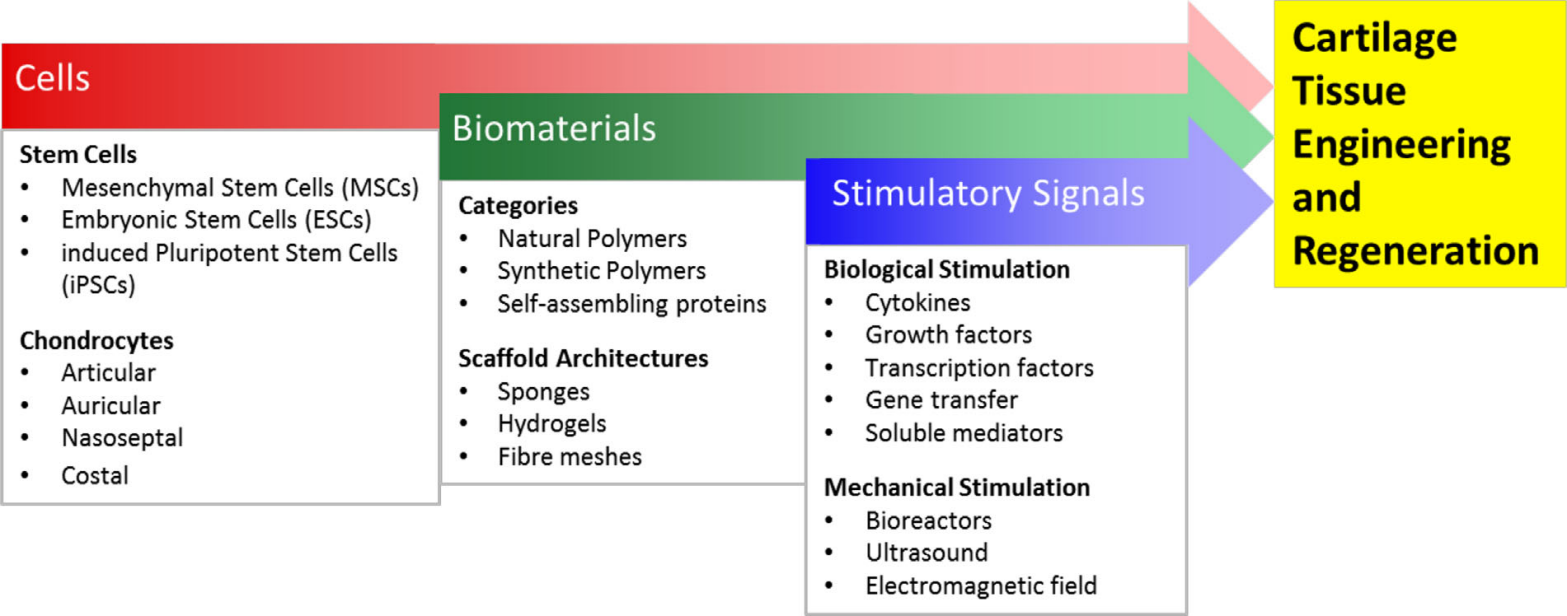
The cartilage regeneration triad. Cells, biomaterials, and stimulatory signals (biological and mechanical) are the main elements currently under investigation for cartilage tissue engineering. Each element has multiple avenues for research, with current cartilage regenerative strategies utilising aspects from each element in isolation or in combination.

The strategies involved in cartilage regeneration can be thought of as a triad.^13^ Cells, including stem cells (mesenchymal, embryonic, and induced pluripotent) and chondrocytes, are a key element of regenerative medicine in addition to the two other important elements; biomaterials for promoting cell growth and construct stability, and stimulatory signals (biological and mechanical) to enhance chondrogenesis (Figure 2). This article will review the potential of mesenchymal stem cell (MSC) populations for cartilage regeneration and outline the roles of biomaterials and stimulatory signals in the cartilage regenerative medicine triad. Furthermore, the surgical treatments for osteochondral lesions of the ankle (OCLA) will be reviewed succinctly to illustrate how the elements of the regenerative triad are currently applied in clinical practice, followed by a discussion of the current challenges for cartilage regeneration, potential solutions, and safety concerns.

## 2 Stem Cell Populations for Cartilage Regeneration

The regenerative potential of stem cells has been studied for over 50 years.^14^, ^15^. Stem cells are characterized by two key features: i) the ability for perpetual or prolonged self‐renewal under controlled conditions and ii) differentiation into multiple cell lineages.^16^, ^17^ From the perspective of cartilage regenerative medicine, human MSCs, induced pluripotent stem cells (iPSCs) and embryonic stem cells (ESCs) are the primary stem cell populations of interest.^18^, ^19^ Human ESCs and iPSCs have been shown to promote cartilage repair in a murine model,^20^, ^21^ however, ethical concerns related to the harvesting of human ESCs and the risk of teratoma formation could prove unsurmountable hurdles to universal acceptance.^22^

MSCs have been reported to be sourced from several tissues, including bone marrow, adipose tissue, synovium, muscle, periosteum, and various connective tissues.^23^, ^24^ MSCs from bone marrow stromal tissue, specifically referred to as skeletal stem cells (SSCs),^25^, ^26^ have attracted much attention in regenerative medicine given their potential to differentiate into chondrocytes upon stimulation of chondrogenesis under appropriate culture conditions. The term SSC defines, specifically, a self‐renewing stem cell that resides in postnatal bone marrow and can differentiate into cartilage, bone, hematopoiesis‐supportive stroma, and marrow adipocytes.^25^, ^26^ However, it is acknowledged that the term MSC, originally applied to a hypothetical common progenitor of a wide range of “mesenchymal” (non‐hematopoietic, non‐epithelial, mesodermal) tissues, is commonly used in published literature to refer to the bone marrow derived stem cell population and will be retained in this review. For clarity, in this review, the prefix b/s/a has been added to MSCs to specifically denote the bone marrow stromal, synovial, and adipose tissue origin of the MSC populations, respectively.

The role of MSCs in cartilage regenerative medicine originates from the observation that bone marrow stromal cells (BMSCs), isolated via the physicochemical property of tissue culture plastic adherence, are able to differentiate into the principal stromal lineages, including chondrocytes.^27^ The tissue culture plastic‐adherent BMSC population is heterogeneous and comprises of bone marrow‐derived MSCs (bMSCs) and osteoprogenitor cells that display differences in their differentiation capacity.^28^ Established stem cell markers have, therefore, been used to isolate discrete populations of MSCs from bone marrow, synovial membranes and adipose tissues for cartilage generation.^29^, ^30^, ^31^, ^32^, ^33^, ^34^, ^35^ However, in the absence of a specific/unique marker for MSCs, there is a lack of consensus in the field regarding which MSC population exhibits superior chondrogenic potential.

The HSC70 antigen, recognized by the STRO‐1 antibody,^36^ is an established marker for MSCs that is also expressed by human articular chondrocytes,^37^ however, STRO‐1^+^ bMSCs exhibit poor chondrogenesis when compared to patient‐matched human articular chondrocytes.^31^ STRO‐4^+^ bMSCs have demonstrated promising in vitro chondrogenic potential, but this observation has yet to be substantiated in vivo.^32^ Human cluster of differentiation (CD) 271^+^ bMSCs were shown to exhibit superior chondrogenic potential in comparison to plastic adherent BMSCs in a murine model.^29^ Moreover, CD271^+^ synovial‐derived MSCs (sMSCs) demonstrated superior chondrogenesis in comparison to CD73^+^ and CD106^+^ sMSCs.^34^ CD105^+^ adipose‐derived MSCs (aMSCs) exhibited robust chondrogenesis in vitro,^35^ however, a high level of CD29 expression in CD105^+^ bMSCs was shown to be crucial for chondrogenic differentiation.^30^ Positive expression of CD56 enhanced the clonogenic efficiency of CD271^bright^ bMSCs and an improved chondrogenic differentiation capacity was observed in CD56^+^/mesenchymal stem cell antigen‐1^+^ bMSCs.^33^

A further challenge in the application of MSCs is related to their frequency within the tissues of origin. The use of bMSCs can be limited by their frequency within bone marrow, which has been reported to be as low as 0.001%.^38^ aMSCs can be harvested through lipoaspiration in greater numbers than bMSCs,^23^ however, aMSCs have been shown to exhibit a lower potential for chondrogenesis.^23^ Alternatively, sMSCs can be isolated in greater numbers than bMSCS or aMSCs^23^ during arthroscopic surgery, which carries a low risk to the patient.^39^ Moreover, bMSCs undergoing chondrogenesis have a propensity for hypertrophy/terminal differentiation and upregulation of hypertrophic markers, namely alkaline phosphatase and type X collagen.^28^, ^31^ In contrast to bMSCs, sMSCs do not exhibit a propensity for hypertrophy^40^ and display high proliferative and chondrogenic properties, which make these cells particularly attractive for cartilage regeneration.^23^

The identity of a discrete MSC population with robust chondrogenic potential and the ability to generate hyaline cartilage analogous to native articular cartilage remains elusive to‐date. It is, therefore, crucial to undertake a comprehensive characterization of the chondrogenic differentiation potential of the diverse MSC populations from multiple tissues, along with a thorough assessment of their ability to generate hyaline cartilage and propensity for hypertrophic differentiation.

## 3 Biomaterials for Cartilage Regenerative Medicine Strategies

Biomaterials retain a promising future in regenerative medicine as they offer significant key attributes: increased control of the tissue microstructure in three dimensions that can be tailored to mimic the native tissue, improved defect filling and the generation of a more stable construct allowing for a shorter postoperative recovery.^41^ Over the last two decades, there has been a rapid rise in the number of commercially available biomaterial products. Biomaterials for cartilage regeneration are grouped into three main categories: i) natural polymers, such as alginate and collagen; ii) synthetic polymers, such as poly‐lactic acid and polyurethane; and iii) self‐assembling peptides.^24^, ^42^ These biomaterials have been utilized to engineer different scaffold architectures, such as sponges,^43^ hydrogels,^44^ and fiber meshes.^45^

A challenge for engineering a scaffold capable of effective chondro and osseointegration lies in the distinct properties of AC compared to bone. AC has a naturally viscoelastic structure and a different biological environment in comparison to stiff, mineralized bone. Furthermore, the structure of the osteochondral interface is also distinct. For the regeneration of osteochondral defects, a biomaterial needs to encompass properties which account for these differences and, therefore, the ideal material should be: porous, bioactive, biocompatible, biodegradable, biomimetic, flexible, elastic, osteoconductive, non‐cytotoxic, non‐antigenic, and have a surface topography which is conducive for cell adhesion, proliferation, and differentiation.^41^, ^46^ Rapid advances in material engineering, cellular biology, and bioengineering offer real opportunities for the development of scaffolds which offer these properties^47^, ^48^ and give surgeons the potential for an unprecedented toolkit for treating OCLA.^49^ What may become difficult in the future, therefore, is not obtaining a biomaterial but deciding which one to use.

There is extensive literature on the application of biomaterials in vitro, yet, reports of clinical data are only slowly emerging and are typically in relation to the knee joint. The low number of clinical studies may be due to lack of product licensing. For example, despite the large numbers of synthetic biomaterials available, currently only a few have been approved for the European Medicines Agency (EMA) or United States Food and Drug Association (FDA) licence (Table 1). As an example, BST‐Cargel® is one product that has approval in Europe and is currently undergoing clinical trials prior to approval in the United States. BST‐Cargel is a soluble polymer scaffold made from chitosan, which is a prevalent glucosamine polysaccharide originating from the exoskeleton of crustaceans. A randomized controlled trial involving 88 knees compared bone marrow stimulation alone with bone marrow stimulation in combination with BST‐CarGel.^50^ Magnetic resonance imaging (MRI) at 1 year showed statistically superior findings in the BST‐CarGel group compared to bone marrow stimulation alone group. However, at this short follow‐up period, no difference was found in the clinical outcome between the two groups. Despite their promise, the clinical findings of BST‐CarGel and other scaffolds such as collagen I/III matrices^51^, ^52^ and Maioregen®^48^, ^53^ are unreliable. Therefore, more biomaterials will need to be licensed and clinically examined before the evidence‐base is sufficient to support their mainstream adoption.

**Table 1:** Products used in cartilage tissue engineering that have either European Medicines Agency (EMA) or Food and Drug Administration (FDA) approval

Trade name	Marketing company	Product components	Regenerative triad elements	Approval authority	Date of approval
		Polyethylene glycol (PEG)	Scaffold	FDA	Pre 2010
		Polylactic acid (PLA)	Scaffold	FDA	Pre 2010
		Polylactide‐glycolic acid (PLGA)	Scaffold	FDA	Pre 2010
Carticel®	Vericel	Autologous chondrocytes	Cells	FDA	Pre 2010
SaluCartilage™	Salumedica	Poly‐vinyl alcohol (PVA) hydrogel	Scaffold	EMA, FDA	Pre 2010
CaReS®	Arthrokinetics	Autologous chondrocytes and type 1 collagen	Cells and scaffold	EMA	Pre 2010
MACI®	Vericel	Autologous chondrocytes and porcine collagen	Cells and scaffold	FDA	Post 2010
BST‐Cargel®	Smith & Nephew	Chitosan polysaccharide	Liquid scaffold	EMA	Post 2010
GelrinC	Reagentis Biomaterials	Polyethylene glycol diacrylate (PEG‐DA) and denatured fibrinogen, crosslinked with UVA light	Scaffold	EMA	Post 2010
Agili‐C™	CartiHeal	Aragonite and hyaluronic acid	Biphasic scaffold	EMA	Post 2010

## 4 Stimulatory Signals for Chondrogenesis

### 4.1 Biological Stimuli

Normal cartilage homeostasis is controlled by transcription factors, cytokines, growth factors, and other environmental cues.^41^ For cartilage regeneration, a number of key candidates have been identified, including the sex‐determining region Y‐type high mobility group box (SOX) transcription factor trio of 5, 6, and 9,^54^ transforming growth factor beta (TGF‐β), and bone morphogenetic protein 2 (BMP‐2).^55^ MSCs can be exposed to the stimulatory factors individually or synergistically to induce and promote chondrogenesis. Chim et al. have shown in a murine model that stromal‐cell‐derived factor 1‐alpha can be combined with TGF‐β1 or BMP‐2 to enhance cartilage repair.^55^ Furthermore, TGF‐β1 and BMP‐2 have been loaded on to a bilaminar scaffold to promote cartilage repair in a lapine model.^56^ Parathyroid hormone‐related protein (PTHrP) was identified as a key factor for chondrogenesis and the minimization of hypertrophy when bMSCs were co‐cultured with human articular chondrocytes.^57^ The application of PTHrP, specifically isoform 1‐34, has, therefore, been advocated to suppress chondrocyte hypertrophy and enhance chondrogenesis.^58^

In addition to responding to exogenous stimulatory factors, MSC can secrete biologically active molecules that have a paracrine effect on other cells.^59^ The paracrine effects can be categorized as trophic (supportive, anti‐apoptotic, and angiogenic), immunomodulatory, anti‐scarring and chemoattractant, and are being increasingly recognized as vital to the success of MSC regeneration.^60^ Exosomes have been identified as the primary vehicle for MSC paracrine secretion^61^ and, promisingly, weekly intra‐articular injections of human embryonic MSC‐derived exosomes have been shown to enhance the repair of osteochondral defects in rats.^62^ Further research is required to validate the potency of exosomes in a large animal model and the clinic, which could lead to the development an “off‐the‐shelf” product that has the advantage of being cell‐free.

The genetic modification of MSCs using viral or non‐viral vectors has also emerged as a potential technique for chondrogenic enhancement.^63^ MSCs transfected with BMP‐2 and SOX‐9 have been reported to promote chondrogenesis in murine models.^64^, ^65^ Additionally, the genetic manipulation of microRNAs in human MSCs has been shown to promote chondrogenesis.^66^ Strategies using genetically manipulated cells offer significant potential, however, a future challenge will be to ensure an appropriate safety profile while still preserving clinical efficacy.^63^

### 4.2 Mechanical Stimuli

Articular cartilage is highly sensitive to its mechanical environment. Mechanical stimulation, therefore, is one of the most important physical cues for improving the biomechanical properties of tissue‐engineered cartilage. Mechanical stimulation can be applied through dynamic compression and shear forces, which when combined, have been shown to promote chondrogenic gene expression in human MSCs seeded on to a polyurethane scaffold.^67^ The biological response of MSCs to the stiffness of the extracellular matrix, or a biomaterial, is an important determining factor for chondrogenesis. Lower substrate stiffness has been shown to promote the expression of chondrogenic genes, namely *SOX9*, *ACAN*, *COMP*, and *COL2*.^68^ Chondrogenic markers were also found to be increased on softer substrates in the presence of TGF‐β.^69^ Additionally, pulsed electromagnetic fields have been reported to promote chondrogenesis in sheep^70^ and a randomized controlled trial of 30 patients using pulsed electromagnetic fields and bMSCs to repair OCLA showed a superior clinical outcome in the trial group over the control group at 12 months’ follow‐up.^71^

It is widely recognized that 3‐D cell culture promotes chondrogenesis,^72^ however, 3‐D culture under static conditions does not promote robust cartilage generation due to the mass transport limitations for oxygen, nutrients, and metabolites.^73^ Bioreactors are dynamic systems that can apply mechanical loading regimens to cells or cell‐seeded biomaterial constructs and are able to replicate the natural physiological cellular environment to promote robust cartilage formation. Several types of bioreactors have been shown to promote chondrogenesis, including mixing flasks,^74^ rotatory flasks,^75^ perfusion bioreactors,^76^ and acoustofluidic perfusion chambers.^77^ A microfluidic, dual chamber bioreactor with separate chondrogenic and osteogenic microenvironments was fabricated to investigate the physiology of human bone marrow stem cell‐derived osteochondral tissue constructs, elucidate the pathogenesis of OA and model tissue responses to potential disease‐modifying OA drugs.^78^ To date, the clinical application of stimulatory signals remains at an early stage due to incomplete understanding of the mechanisms at play. Thus, further research is needed to improve our understanding of the role of stimulatory signals in AC regeneration.

## 5 Current Surgical Regenerative Strategies for the Treatment of OCLA

A large number of in vitro and in vivo animal studies have been undertaken to identify promising avenues for cartilage regeneration. However, a key test for laboratory‐based research is the ability to progress from the bench to the clinic and, despite the progress made using in vivo animal models,^79^, ^80^ the use of human allogenic MSC populations for cartilage regeneration has only just started.^81^ Outlined below are the current surgical treatments for OCLA that employ autologous BMSC and MSC populations with or without the inclusion of biomaterials or stimulatory signals.

### 5.1 Bone Marrow Stimulation

**Figure 3:**
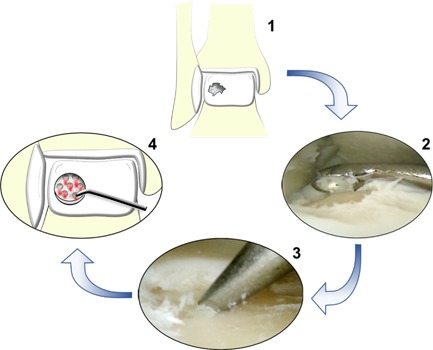
An illustration of the microfracture technique for the treatment of osteochondral lesion of the ankle. 1) A full‐thickness cartilage lesion is prepared by debriding the damaged cartilage. 2) The calcified cartilage from the base of the lesion is removed using a curette. 3) Perforations in the subchondral bone are made every 3–4 mm using a metal pick. 4) The perforations allow bone marrow components to enter the chondral defect and form a coagulate, which leads to the formation of fibrocartilage.

Even though specific MSC sub/populations have yet to enter mainstream clinical trials, unselected BMSCs have been applied for cartilage regeneration since the 1950s and form the basis of many current surgical treatments. In a technique known as bone marrow stimulation (BMS), BMSCs are delivered into AC defects by accessing the medullary cavity of the long bones. This is a cost‐effective and relatively simple procedure, achieved by either drilling through the subchondral bone into the medullary cavity, known as Pridie drilling,^82^ or forcing a metal pick through the subchondral bone, known as microfracture^83^ (Figure 3). Once the subchondral bone is breached, bone marrow components enter the joint and a coagulate forms within the chondral defect. The coagulate contains progenitor cells, BMSCs and the extracellular components required for healing, leading to the formation of fibrocartilage instead of hyaline cartilage, which is the main limitation in bone marrow stimulation. Fibrocartilage consists predominately of type I collagen as opposed to the hyaline cartilage‐specific type II collagen and, therefore, has inferior biomechanical properties compared to native AC.^84^ Due to its inferior structure, fibrocartilage has been found to breakdown quickly leading to fibrillation and deterioration after only a few years.^85^

Due to the vulnerability of fibrocartilage, there is conflicting evidence in the literature on the role of BMS in the repair of OCLA.^86^, ^87^ Age, duration of symptoms, and a traumatic aetiology have not been found to influence outcome^88^ but superior results seem to be associated with smaller lesions.^89^ Therefore, BMS is considered a suitable primary treatment for OCLA that are less than 150 mm^2^.^90^

### 5.2 Autologous Matrix‐Induced Chondrogenesis

In a bid to overcome the shortcomings of BMS, the technique of releasing BMSCs into a chondral defect can be combined with a biomaterial. An osteochondral lesion is prepared in a similar manner to BMS, and if a subchondral cyst is present, the bone defect can be filled with cancellous bone from the iliac crest, a recognized, viable source of BMSCs.^91^ Once the lesion has been prepared, the biomaterial is cut to size using a template and glued into the defect using fibrin. After the operation, a coagulate forms within the biomaterial leading to chondrogenesis. Two biomaterials used in recent publications are a solid, acellular collagen I/III matrix of porcine origin^51^ and MaioRegen®, a solid collagen I and magnesium‐hydroxyapatite matrix.^48^, ^53^ The matrices are bilaminar and trilaminar, respectively, consisting of a compact upper construct that contains the blood coagulate within the defect and a porous lower side composed of loose collagen fibers to support chondrogenesis and osteogenesis.

A clear advantage of autologous matrix‐induced chondrogenesis is its one‐step and “off‐the‐shelf” nature. Nevertheless, the short‐term clinical results for treating OCLA have been poor, with short‐term follow‐up showing inconsistent tissue regeneration, hypertrophic cartilage, and high treatment failure rates.^48^, ^51^, ^53^ As early results are not encouraging, it remains too early to confirm if this method of integrating biomaterials with BMSCs will become truly validated.

### 5.3 Bone Marrow Aspirate Concentrate

Bone marrow aspirate concentrate (BMAC) is a technique that utilizes the plasma and bone marrow mononuclear cells from freshly aspirated bone marrow without the granulocyte or anuclear, erythrocyte fractions. The technique involves aspirating 60 milliliters of bone marrow from the anterior or posterior iliac spines. After aspiration, the mononuclear cells are isolated in the operating theater via differential centrifugation. Given there is no further attempt to isolate a homogenous population of bMSCs from the mononuclear cells, the theoretical grounding for this technique is poor as the fraction of bMSCs isolated via this method may be as low as 0.01–0.001%.^38^

BMAC has been used in combination with a variety of different biomaterials for treating OCLA but results have been mixed.^92^, ^93^ A systematic review of 184 patients from four studies found a significant improvement in the post‐operative clinical scores at a mean of 2.86 years’ follow‐up.^94^ However, MRI findings at four years’ follow‐up revealed incomplete defect filling in 55% of patients.^93^ The inconsistent results and scarcity of evidence for this technique mean that definitive conclusions cannot currently be drawn.

### 5.4 Bone Marrow Stimulation Adjuncts

An adjunct to BMS is the use of platelet‐rich plasma (PRP) or, the second‐generation platelet concentrate, platelet‐rich fibrin (PRF).^95^ Platelets are concentrated approximately fivefold and the increased platelet count delivers a higher than normal concentration of growth factors, such as platelet‐derived growth factor and vascular endothelial growth factor, to the area of treatment.^96^ In theory, the growth factors are then able to increase the stimulation of the BMSCs to enhance chondrogenesis.^97^ The platelet concentrate may be used in conjunction with a biomaterial and BMAC^93^ or injected postoperatively.^98^

Only a few studies have been published evaluating the role of PRP in the treatment of OCLA and a systematic review, including 268 patients in seven studies, advised caution over its use.^99^ A prospective trial with short follow‐up has shown that hyaluronic acid is a potential alternative BMS adjunct^100^ but further conclusive evidence is lacking.

### 5.5 Mesenchymal Stem Cell Injection

The intra‐articular injection of MSCs is an emerging technique with the theoretical grounding based on the trophic, homing and immunomodulatory properties of MSCs.^101^ To date, only a few studies have been published relating to OCLA and all are from the same team in South Korea.^102^, ^103^, ^104^ The authors harvested autologous aMSCs from gluteal fat the day before surgery and then injected the aMSCs after BMS surgery to treat OCLA. At a maximum of 2.3 years’ follow‐up, significant clinical outcomes were achieved in the aMSC injection group compared to the control group. However, despite these promising results, one of the studies reported several complications at the follow‐up arthroscopy of 62 ankles.^104^ 98% of the ankles required a debridement, 60% required a synovectomy, and 52% required removal of intraarticular adhesions. Despite positive short‐term clinical results, clearly, further research from other groups is required to validate this treatment in the future.

## 6 Current Challenges and Potential Solutions

Although considerable progress has been made in the field of cartilage regeneration, there are many challenges that need to be addressed. Cell‐based strategies are hindered by a lack of consensus within the field regarding the most suitable cell source for cartilage regeneration. Issues regarding donor‐to‐donor variability, MSC heterogenicity, the propensity of bMSC‐derived chondrocytes for hypertrophic differentiation, and dedifferentiation of chondrocytes in monolayer cultures need to be resolved. Moreover, cell potency and viability need to be maintained throughout the multistage clinical application process.

A possible solution to overcome some of these challenges is through the identification of the, as yet, elusive population of MSCs characterised by robust chondrogenic potential, combined with the application of improved chemical and temporal differentiation protocols that support reliable chondrogenesis in the cell population of choice. The identification of a consistent cell source, through hybrid co‐culture, ESCs, iPSCs, or allogenic MSCs, could minimize variability and heterogenicity, and facilitate the development of simplified one‐step surgical techniques. Furthermore, bioengineering technologies that provide enhanced structural support as well as mechanical and biological stimulation to the cells or developing cartilage tissue are likely to be crucial for future strategies and facilitate the manufacture of “off‐the‐shelf” products.

Native AC has a complex functional stratification, with each zone exhibiting different properties and performing different physiological roles. To date, cartilage regeneration strategies attempting to replicate the zonal structure of native AC have achieved limited success. Approaches for recapitulating the stratified zonal architecture of native AC and its important physiological characteristics, such as superficial zone protein/lubricin secretion, include utilisation of zone‐specific chondrocytes and MSCs derived from the synovium and infrapatellar fat pad.^105^, ^106^, ^107^

Moreover, as each element of the regenerative triad is optimized, the process of combining the three elements will also need optimization. 3‐D bioprinting offers the potential to combine the three elements of the regenerative triad during the manufacturing process, as opposed to each element being applied individually. The technique involves printing layer‐by‐layer, not only a biomaterial but also all the appropriate biological constituents, including the living cells and proteins required for tissue regeneration.^108^ The particular advantage of this approach is that the distribution of cells and stimulatory signals within the 3‐D bioprinted material can be precisely controlled resulting in a more homogenous construct.^109^ Additionally, the digital nature of the process allows patient‐specific tissue constructs and treatments to be created from clinical diagnostic images.^110^ The rapidly expanding 3‐D bioprinting technology has been applied to print human chondrocytes for repairing defects in osteochondral plugs in vitro.^111^

## 7 Safety

The emerging field of regenerative medicine has many unknowns relating to the safe use of MSC populations and biological adjuncts. There is some evidence that MSCs are immunoprivileged, immunosuppressive, and do not induce tumor development,^112^, ^113^ and commercially available porcine collagen matrices have been shown to be safe and beneficial.^51^, ^114^ Furthermore, to date, there have been no published reports of tumor formation in patients where chondrocytes or MSCs have been used. However, the apparent low complication rate associated with regenerative medicine agents may lead to a false sense of security and caution should be maintained for all new products. For instance, Hyalograft C, a biomaterial used in autologous chondrocyte implantation, has recently been withdrawn from the European market due to safety concerns over its manufacture.^115^

In the face of current clinical evidence, although limited, stem cell treatment appears to be promising and safe. To date, two groups have published systematic reviews that analyzed the safety of stem cell treatments in patients with a range of orthopaedic and medically‐related diseases.^116^, ^117^ The authors looked at a wide range of adverse events including infection, malignancy, and death but only found a significant correlation between stem cell treatment and transient fever, increased joint pain, and swelling. Despite this favorable finding, there remains an urgent need for an improved safety and clinical evidence base.

## 8 Conclusions

Although regenerative medicine has progressed a long way from its inception over 40 years ago, cartilage regeneration remains a challenge due to a continued lack of consensus in the field regarding the most effective clinical application utilizing cells, biomaterials, and stimulatory signals. Novel techniques and commercially available products will undoubtedly become part of the mainstream treatment for OCLA in the imminent future, but, in comparison to the knee, regenerative strategies in the ankle are relatively new and lack randomized control trials to validate treatment efficacy. Furthermore, as of September 2017, there are only seven active clinical trials registered on the website clinicaltrials.org relating to OCLA. This is a cause for concern and may reflect the difficulty in transferring pre‐clinical success to clinical trials.

Moreover, whilst continual advancement is good for the patient, this can be hard for the surgeon. With no technique showing significantly better results, it can be confusing for the surgeon to decide which technique should be used for each clinical problem. Treatment algorithms for OCLA^118^ can help crystallize possible treatment pathways, however, until there are convincing evidence‐based treatments, surgeons will remain in the dark about which is the best and most cost‐effective way of using MSC populations and/or chondrocytes to treat their patients.

## Conflicts of Interest

The authors declare no commercial and financial conflicts of interest.

## References

[1] W. Hunter , Philo. Trans. Royal Soc. London 1743, 42b, 514.

[2] J. W. J. Bijlsma , F. Berenbaum , F. P. Lafeber , Lancet 2011, 377, 2115. 21684382 10.1016/S0140-6736(11)60243-2

[3] A. Litwic , M. H. Edwards , E. M. Dennison , C. Cooper , Br. Med. Bull. 2013, 105, 185. 23337796 10.1093/bmb/lds038 PMC3690438

[4] C. Mason , P. Dunnill , Regen. Med. 2008, 3, 1. 18154457 10.2217/17460751.3.1.1

[5] S. L. Jensen , B. K. Andresen , S. Mencke , P. T. Nielsen , Acta Orthop. Scand. 2009, 69, 48. 10.3109/17453679809002356 9524518

[6] N. Leontaritis , L. Hinojosa , V. K. Panchbhavi , J. Bone Joint Surg. Am. Vol. 2009, 91, 333. 10.2106/JBJS.H.00584 19181977

[7] M. Brittberg , A. Lindahl , A. Nilsson , C. Ohlsson , O. Isaksson , L. Peterson , New Engl. J. Med. 1994, 331, 889. 8078550 10.1056/NEJM199410063311401

[8] S. K. Kwak , B. S. Kern , R. D. Ferkel , K. W. Chan , S. Kasraeian , G. R. Applegate , Am. J. Sports Med. 2014, 42, 2156. 25056988 10.1177/0363546514540587

[9] P. Niemeyer , G. Salzmann , H. Schmal , H. Mayr , N. P. Sudkamp , Knee Surg. Sports Traum. Arthros. 2012, 20, 1696. 10.1007/s00167-011-1729-0 22037894

[10] J. Dominice , C. Levasseur , S. Larno , X. Ronot , M. Adolphe , Mechan. Age. Dev. 1986–**1987**, 37, 231. 10.1016/0047-6374(86)90040-0 3553761

[11] P. D. Benya , J. D. Shaffer , Cell 1982, 30, 215. 7127471 10.1016/0092-8674(82)90027-7

[12] M. Thomas , M. Jordan , E. Hamborg‐Petersen , Der Unfallchirurg 2016, 119, 100. 26810230 10.1007/s00113-015-0136-2

[13] F. J. O'Brien , Mater. Today 2011, 14, 88.

[14] E. A. McCulloch , J. E. Till , Radiat. Res. 1960, 13, 115. 13858509

[15] A. J. Friedenstein , I. I. Piatetzky‐Shapiro , K. V. Petrakova , J. Embryol. Exp. Morph. 1966, 16, 381. 5336210

[16] A. J. Friedenstein , R. K. Chailakhjan , K. S. Lalykina , Cell Tissue Kinetics 1970, 3, 393. 5523063 10.1111/j.1365-2184.1970.tb00347.x

[17] H. Castro‐Malaspina , R. E. Gay , G. Resnick , N. Kapoor , P. Meyers , D. Chiarieri , S. McKenzie , H. E. Broxmeyer , M. A. Moore , Blood 1980, 56, 289. 6994839

[18] M. Baghaban Eslaminejad , E. Malakooty Poor , World J. Stem Cells 2014, 6, 344. 25126383 10.4252/wjsc.v6.i3.344 PMC4131275

[19] W. S. Toh , Pluripotent stem cells: differentiation potential and therapeutic efficacy for cartilage repair In Pluripotent Stem Cells − From the Bench to the Clinic, Ed: Tomizawa M., InTech, Intech, Rijeka, Croatia 2016.

[20] A. Cheng , Z. Kapacee , J. Peng , S. Lu , R. J. Lucas , T. E. Hardingham , S. J. Kimber , Stem Cells Trans. Med. 2014, 3, 1287. 10.5966/sctm.2014-0101 PMC4214847 25273540

[21] A. Yamashita , M. Morioka , Y. Yahara , M. Okada , T. Kobayashi , S. Kuriyama , S. Matsuda , N. Tsumaki , Stem Cell Rep. 2015, 4, 404. 10.1016/j.stemcr.2015.01.016 PMC4375934 25733017

[22] M.‐O. Lee , S. H. Moon , H.‐C. Jeong , J.‐Y. Yi , T.‐H. Lee , S. H. Shim , Y.‐H. Rhee , S.‐H. Lee , S.‐J. Oh , M.‐Y. Lee , M.‐J. Han , Y. S. Cho , H.‐M. Chung , K.‐S. Kim , H.‐J. Cha , Proc. Natl. Acad. Sci. USA 2013, 110, E3281. 23918355 10.1073/pnas.1303669110 PMC3761568

[23] Y. Sakaguchi , I. Sekiya , K. Yagishita , T. Muneta , Arthritis Rheum. 2005, 52, 2521. 16052568 10.1002/art.21212

[24] C. Chung , J. A. Burdick , Adv. Drug Deliv. Rev. 2008, 60, 243. 17976858 10.1016/j.addr.2007.08.027 PMC2230638

[25] P. Bianco , P. G. Robey , P. J. Simmons , Cell Stem Cell 2008, 2, 313. 18397751 10.1016/j.stem.2008.03.002 PMC2613570

[26] R. S. Tare , J. C. Babister , J. Kanczler , R. O. C. Oreffo , Mol. Cell. Endocrinol. 2008, 288, 11. 18395331 10.1016/j.mce.2008.02.017

[27] M. Owen , A. J. Friedenstein , Ciba Foundation Symp. 1988, 136, 42. 10.1002/9780470513637.ch4 3068016

[28] S. Gronthos , A. C. Zannettino , S. J. Hay , S. Shi , S. E. Graves , A. Kortesidis , P. J. Simmons , J. Cell Sci. 2003, 116, 1827. 12665563 10.1242/jcs.00369

[29] Y. Mifune , T. Matsumoto , S. Murasawa , A. Kawamoto , R. Kuroda , T. Shoji , T. Kuroda , T. Fukui , Y. Kawakami , M. Kurosaka , T. Asahara , Cell Trans. 2013, 22, 1201. 10.3727/096368912X657378 23044363

[30] C. Cicione , S. Díaz‐Prado , E. Muiños‐López , T. Hermida‐Gómez , F. J. Blanco , ; Res. Biol. Div. 2010, 80, 155. 10.1016/j.diff.2010.06.001 20619527

[31] S. Li , B. G. Sengers , R. O. C. Oreffo , R. S. Tare , J. Biomater. Appl. 2015, 29, 824. 25145989 10.1177/0885328214548604 PMC4274334

[32] S. Gronthos , R. McCarty , K. Mrozik , S. Fitter , S. Paton , D. Menicanin , S. Itescu , P. M. Bartold , C. Xian , A. C. W. Zannettino , Stem Cells Dev. 2009, 18, 1253. 19327008 10.1089/scd.2008.0400

[33] V. L. Battula , S. Treml , P. M. Bareiss , F. Gieseke , H. Roelofs , P. d. Zwart , I. Müller , B. Schewe , T. Skutella , W. E. Fibbe , L. Kanz , H.‐J. Bühring , Haematologica 2009, 94, 173. 19066333 10.3324/haematol.13740 PMC2635396

[34] M. C. Arufe , A. De la Fuente , I. Fuentes , F. J. de Toro , F.J Blanco , J. Cell. Biochem. 2010, 111, 834. 20665538 10.1002/jcb.22768

[35] T. Jiang , W. Liu , X. Lv , H. Sun , L. Zhang , Y. Liu , W. J. Zhang , Y. Cao , G. Zhou , Biomaterials 2010, 31, 3564. 20153525 10.1016/j.biomaterials.2010.01.050

[36] S. Fitter , S. Gronthos , S. S. Ooi , A. C. W. Zannettino , Stem Cells 2016, 35, 940. 10.1002/stem.2560 28026090

[37] S. P. Grogan , S. Miyaki , H. Asahara , D. D. D'Lima , M. K. Lotz , Arthritis Res. Ther. 2009, 11, R85. 19500336 10.1186/ar2719 PMC2714136

[38] M. F. Pittenger , A. M. Mackay , S. C. Beck , R. K. Jaiswal , R. Douglas , J. D. Mosca , M. A. Moorman , D. W. Simonetti , S. Craig , D. R. Marshak , Science 1999, 284, 143. 10102814 10.1126/science.284.5411.143

[39] J. Fan , R. R. Varshney , L. Ren , D. Cai , D.‐A. Wang , Tissue Eng. Part B Rev. 2009, 15, 75. 19196118 10.1089/ten.teb.2008.0586

[40] B. A. Jones , M. Pei , Tissue Eng. Part B Rev. 2012, 18, 301. 22429320 10.1089/ten.TEB.2012.0002

[41] J. M. Oliveira , R. L. Reis , Regenerative Strategies for the Treatment of Knee Joint Disabilities. Springer Berlin Heidelberg, New York, NY 2016.

[42] T. C. Holmes , Trends Biotechnol. 2002, 20, 16. 11742673 10.1016/s0167-7799(01)01840-6

[43] N. J. Goodstone , A. Cartwright , B. Ashton , Tissue Eng. 2004, 10, 621. 15165478 10.1089/107632704323061979

[44] H. J. Lee , J.‐S. Lee , T. Chansakul , C. Yu , J. H. Elisseeff , S. M. Yu , Biomaterials 2006, 27, 5268. 16797067 10.1016/j.biomaterials.2006.06.001

[45] H. J. Shin , C. H. Lee , I. H. Cho , Y.‐J. Kim , Y.‐J. Lee , I. A. Kim , K.‐D. Park , N. Yui , J.‐W. Shin , J. Biomater. Sci. Polym. Ed. 2006, 17, 103. 16411602 10.1163/156856206774879126

[46] E. G. Khaled , M. Saleh , S. Hindocha , M. Griffin , W. S. Khan , Open Orthop. J. 2011, 2(5 Suppl), 289. 10.2174/1874325001105010289 PMC3149819 21886695

[47] C. J. Pearce , L. E. Gartner , A. Mitchell , J. D. Calder , Foot Ankle Surg. 2012, 18, 114. 22443998 10.1016/j.fas.2011.04.001

[48] B. B. Christensen , C. B. Foldager , J. Jensen , N. C. Jensen , M. Lind , Knee Surg. Sports Traumat. Arthros. 2016, 24, 2380. 10.1007/s00167-015-3538-3 25691368

[49] B. Johnstone , M. Alini , M. Cucchiarini , G. R. Dodge , D. Eglin , F. Guilak , H. Madry , A. Mata , R. L. Mauck , C. E. Semino , M. J. Stoddart , Eur. Cells Mater. 2013, 25, 248. 10.22203/ecm.v025a18 23636950

[50] W. D. Stanish , R. McCormack , F. Forriol , N. Mohtadi , S. Pelet , J. Desnoyers , A. Restrepo , M. S. Shive , J. Bone Joint Surg. Am. Vol. 2013, 95, 1640. 10.2106/JBJS.L.01345 24048551

[51] V. Valderrabano , M. Miska , A. Leumann , M. Wiewiorski , Am. J. Sports Med. 2013, 41, 519. 23393079 10.1177/0363546513476671

[52] M. Wiewiorski , M. Miska , M. Kretzschmar , U. Studler , O. Bieri , V. Valderrabano , Clinical Radiol. 2013, 68, 1031. 10.1016/j.crad.2013.04.016 23809267

[53] D. Albano , N. Martinelli , A. Bianchi , C. Messina , F. Malerba , L. M. Sconfienza , BMC Musculoskeletal Disord. 2017, 18, 306. 10.1186/s12891-017-1679-x PMC5516391 28720091

[54] T. Ikeda , S. Kamekura , A. Mabuchi , I. Kou , S. Seki , T. Takato , K. Nakamura , H. Kawaguchi , S. Ikegawa , U.‐i. Chung , Arthritis Rheumat. 2004, 50, 3561. 15529345 10.1002/art.20611

[55] H. Chim , E. Miller , C. Gliniak , E. Alsberg , Cell Tissue Res. 2012, 350, 89. 22684849 10.1007/s00441-012-1449-x

[56] R. Reyes , A. Delgado , R. Solis , E. Sanchez , A. Hernandez , J. San Roman , C. Evora , J. Biomed. Mater. Res Part A 2014, 102, 1110. 10.1002/jbma.34769 23766296

[57] J. Fischer , A. Dickhut , M. Rickert , W. Richter , Arthritis Rheum. 2010, 62, 2696. 20496422 10.1002/art.27565

[58] J.‐M. Lee , G.‐I. Im , Biochem. Biophys. Res. Commun. 2012, 421, 819. 22554518 10.1016/j.bbrc.2012.04.096

[59] A. I. Caplan , J. E. Dennis , J. Cell. Biochem. 2006, 98, 1076. 16619257 10.1002/jcb.20886

[60] L. d. S. Meirelles , A. M. Fontes , D. T. Covas , A. I. Caplan , Cytokine Growth Factor Rev. 2009, 20, 419. 19926330 10.1016/j.cytogfr.2009.10.002

[61] W. S. Toh , R. C. Lai , J. H. P. Hui , S. K. Lim , Sem. Cell Dev. Biol. 2017, 67, 56. 10.1016/j.semcdb.2016.11.008 27871993

[62] S. Zhang , W. C. Chu , R. C. Lai , S. K. Lim , J. H. P. Hui , W. S. Toh , Osteoarthritis Cartilage 2016, 24, 2135. 27390028 10.1016/j.joca.2016.06.022

[63] S. Gurusinghe , P. Strappe , BioMed Res. Int. 2014, 2014, 369528. 24963479 10.1155/2014/369528 PMC4052490

[64] T. Zachos , A. Diggs , S. Weisbrode , J. Bartlett , A. Bertone , Mol. Ther. 2007, 15, 1543. 17519894 10.1038/sj.mt.6300192

[65] J. C. Babister , R. S. Tare , D. W. Green , S. Inglis , S. Mann , R. O. C. Oreffo , Biomaterials 2008, 29, 58. 17897711 10.1016/j.biomaterials.2007.09.006

[66] A. Lolli , R. Narcisi , E. Lambertini , L. Penolazzi , M. Angelozzi , N. Kops , S. Gasparini , G. J. V. M. van Osch , R. Piva , Stem Cells 2016, 34, 1801. 26930142 10.1002/stem.2350

[67] O. Schatti , S. Grad , J. Goldhahn , G. Salzmann , Z. Li , M. Alini , M. J. Stoddart , Eur. Cells Mater. 2011, 22, 214. 10.22203/ecm.v022a17 22048899

[68] R. Olivares‐Navarrete , E. M. Lee , K. Smith , S. L. Hyzy , M. Doroudi , J. K. Williams , K. Gall , B. D. Boyan , Z. Schwartz , PLoS ONE 2017, 12, e0170312. 28095466 10.1371/journal.pone.0170312 PMC5240960

[69] J. S. Park , J. S. Chu , A. D. Tsou , R. Diop , Z. Tang , A. Wang , S. Li , Biomaterials 2011, 32, 3921. 21397942 10.1016/j.biomaterials.2011.02.019 PMC3073995

[70] F. Benazzo , M. Cadossi , F. Cavani , M. Fini , G. Giavaresi , S. Setti , R. Cadossi , R. Giardino , J. Orthop. Res. 2008, 26, 631. 18176941 10.1002/jor.20530

[71] M. Cadossi , R. E. Buda , L. Ramponi , A. Sambri , S. Natali , S. Giannini , Foot Ankle Int. 2014, 35, 981. 24917648 10.1177/1071100714539660

[72] Y. Kato , M. Iwamoto , T. Koike , F. Suzuki , Y. Takano , Proc. Natl. Acad. Sci. USA 1988, 85, 9552. 3200840 10.1073/pnas.85.24.9552 PMC282794

[73] S. Li , R. O. C. Oreffo , B. G. Sengers , R. S. Tare , Biotechnol. Bioeng. 2014, 111, 1876. 24668194 10.1002/bit.25241 PMC4284020

[74] G. Vunjak‐Novakovic , L. E. Freed , R. J. Biron , R. Langer , AIChE J. 1996, 42, 850.

[75] L. E. Freed , A. P. Hollander , I. Martin , J. R. Barry , R. Langer , G. Vunjak‐Novakovic , Exp. Cell Res. 1998, 240, 58. 9570921 10.1006/excr.1998.4010

[76] R. W. Forsey , R. Tare , R. O. C. Oreffo , J. B. Chaudhuri , Biotechnol. Appl. Biochem. 2012, 59, 142. 23586794 10.1002/bab.1009

[77] S. Li , P. Glynne‐Jones , O. G. Andriotis , K. Y. Ching , U. S. Jonnalagadda , R. O. C. Oreffo , M. Hill , R. S. Tare , Lab Chip 2014, 14, 4475. 25272195 10.1039/c4lc00956h PMC4227593

[78] H. Lin , T. P. Lozito , P. G. Alexander , R. Gottardi , R. S. Tuan , Mol. Pharm. 2014, 11, 2203. 24830762 10.1021/mp500136b PMC4086740

[79] T. Nakamura , I. Sekiya , T. Muneta , D. Hatsushika , M. Horie , K. Tsuji , T. Kawarasaki , A. Watanabe , S. Hishikawa , Y. Fujimoto , H. Tanaka , E. Kobayashi , Cytotherapy 2012, 14, 327. 22309371 10.3109/14653249.2011.638912 PMC3296518

[80] J.‐C. Lee , S. Y. Lee , H. J. Min , S. A. Han , J. Jang , S. Lee , S. C. Seong , M. C. Lee , Tissue Eng. Part A 2012, 18, 2173. 22765885 10.1089/ten.TEA.2011.0643

[81] T. S. Windt de , L. A. Vonk , I. C. M. Slaper‐Cortenbach , M. P. H. van den Broek , R. Nizak , H. P. van Rijen Mattie , R. A. d. Weger , W. J. A. Dhert , D. B. F. Saris , Stem Cells 2017, 35, 256. 27507787 10.1002/stem.2475

[82] J. Insall , Clinical Orthop. Relat. Res. 1974, 101, 61. 4837919

[83] J. R. Steadman , W. G. Rodkey , J. J. Rodrigo , Clin. Orthop. Relat. Res. 2001 (391 Suppl), S362‐9. 10.1097/00003086-200110001-00033 11603719

[84] F. Shapiro , S. Koide , M. J. Glimcher , J. Bone Joint Surg. Am Vol. 1993, 75, 532. 10.2106/00004623-199304000-00009 8478382

[85] C. Becher , A. Driessen , T. Hess , U. G. Longo , N. Maffulli , H. Thermann , Knee Surg. Sports Traumatol. Arthr. 2010, 18, 656. 10.1007/s00167-009-1036-1 20130840

[86] D. J. Cuttica , J. A. Shockley , C. F. Hyer , G. C. Berlet , Foot Ankle Special. 2011, 4, 274. 10.1177/1938640011411082 21926371

[87] R. D. Ferkel , R. M. Zanotti , G. A. Komenda , N. A. Sgaglione , M. S. Cheng , G. R. Applegate , R. M. Dopirak , Am. J. Sports Med. 2008, 36, 1750. 18753679 10.1177/0363546508316773

[88] W. J. Choi , K. K. Park , B. S. Kim , J. W. Lee , Am. J Sports Med. 2009, 37, 1974. 19654429 10.1177/0363546509335765

[89] L. Ramponi , Y. Yasui , C. D. Murawski , R. D. Ferkel , C. W. DiGiovanni , G. M. M. J. Kerkhoffs , J. D. F. Calder , M. Takao , F. Vannini , W. J. Choi , J. W. Lee , J. Stone , J. G. Kennedy , Am. J. Sports Med. 2016, 45, 1698. 27852595 10.1177/0363546516668292

[90] S. T. Grambart , Clin. Podiatr. Med. Surg. 2016, 33, 521. 27599437 10.1016/j.cpm.2016.06.008

[91] J. W. Canady , D. P. Zeitler , S. A. Thompson , C. D. Nicholas , Cleft Palate‐Craniof. J. 1993, 30, 579. 10.1597/1545-1569_1993_030_0579_sotica_2.3.co_2 8280737

[92] C. P. Hannon , K. A. Ross , C. D. Murawski , T. W. Deyer , N. A. Smyth , M. V. Hogan , H. T. Do , M. J. O'Malley , J. G. Kennedy , Arthroscopy 2016, 32, 339. 26395409 10.1016/j.arthro.2015.07.012

[93] S. Giannini , R. Buda , M. Battaglia , M. Cavallo , A. Ruffilli , L. Ramponi , G. Pagliazzi , F. Vannini , Am. J. Sports Med. 2013, 41, 511. 23221772 10.1177/0363546512467622

[94] J. Chahla , M. E. Cinque , J. M. Shon , D. J. Liechti , L. M. Matheny , R. F. LaPrade , T. O. Clanton , J. Exp. Orthop. 2016, 3, 33. 27813021 10.1186/s40634-016-0069-x PMC5095091

[95] H. Saluja , V. Dehane , U. Mahindra , Ann. Maxill. Surg. 2011, 1, 53. 10.4103/2231-0746.83158 PMC3591032 23482459

[96] R. E. Marx , Implant Dent. 2001, 10, 225. 11813662 10.1097/00008505-200110000-00002

[97] L. A. Fortier , C. H. Hackett , B. J. Cole , Oper. Techn. Sports Med. 2011, 19, 154.

[98] A. Guney , M. Akar , I. Karaman , M. Oner , B. Guney , Knee Surgery Sports Traumatol. Arthr. 2015, 23, 2384. 10.1007/s00167-013-2784-5 24292979

[99] F. Vannini , B. Di Matteo , G. Filardo , J. Exp. Orthop. 2015, 2, 2. 26914870 10.1186/s40634-015-0019-z PMC4546066

[100] M. N. Doral , O. Bilge , G. Batmaz , G. Donmez , E. Turhan , M. Demirel , O. A. Atay , A. Uzumcugil , K. Atesok , D. Kaya , Knee Surgery Sports Traumatol. Arthr. 2012, 20, 1398. 10.1007/s00167-011-1856-7 22205098

[101] X. Wei , X. Yang , Z.‐p. Han , F.‐f. Qu , L. Shao , Y.‐f. Shi , Acta Pharm. Sin. 2013, 34, 747. 10.1038/aps.2013.50 PMC4002895 23736003

[102] Y. S. Kim , E. H. Park , Y. C. Kim , Y. G. Koh , Am. J. Sports Med. 2013, 41, 1090. 23460335 10.1177/0363546513479018

[103] Y. S. Kim , H. J. Lee , Y. J. Choi , Y. I. Kim , Y. G. Koh , Am. J. Sports Med. 2014, 42, 2424. 25106781 10.1177/0363546514541778

[104] Y. S. Kim , M. Lee , Y. G. Koh , J. Exp. Orthop. 2016, 3, 12. 27206975 10.1186/s40634-016-0048-2 PMC4875581

[105] T.‐K. Kim , B. Sharma , C. Williams , M. Ruffner , A. Malik , E. McFarland , J. Elisseeff , Osteoarthritis Cartilage 2003, 11, 653. 12954236 10.1016/s1063-4584(03)00120-1

[106] B. Sharma , C. G. Williams , T. K. Kim , D. Sun , A. Malik , M. Khan , K. Leong , J. Elisseeff , Tissue Eng. 2007, 13, 405. 17504064 10.1089/ten.2006.0068

[107] S. Y. Lee , T. Nakagawa , A. H. Reddi , Tissue Eng. Part A 2010, 16, 317. 19702511 10.1089/ten.TEA.2009.0104

[108] T. Birtchnell , W. Hoyle , 3D Printing for Development in the Global South: The 3D4D Challenge. Palgrave Macmillan, Basingstoke 2014.

[109] X. Cui , G. Gao , T. Yonezawa , G. Dai , J. Vis. Exp. 2014, 88, e51294. 10.3791/51294 PMC4188426 24961492

[110] H.‐W. Kang , S. J. Lee , I. K. Ko , C. Kengla , J. J. Yoo , A. Atala , Nat. Biotechnol. 2016, 34, 312. 26878319 10.1038/nbt.3413

[111] X. Cui , K. Breitenkamp , M. G. Finn , M. Lotz , D. D. D'Lima , Tissue Eng. Part A 2012, 18, 1304. 22394017 10.1089/ten.tea.2011.0543 PMC3360507

[112] S. Aggarwal , M. F. Pittenger , Blood 2005, 105, 1815. 15494428 10.1182/blood-2004-04-1559

[113] J. C. Ra , I. S. Shin , S. H. Kim , S. K. Kang , B. C. Kang , H. Y. Lee , Y. J. Kim , J. Y. Jo , E. J. Yoon , H. J. Choi , E. Kwon , Stem Cells Dev. 2011, 20, 1297. 21303266 10.1089/scd.2010.0466

[114] K. Zaslav , B. Cole , R. Brewster , T. DeBerardino , J. Farr , P. Fowler , C. Nissen , Am. J. Sports Med. 2009, 37, 42. 22822178 10.1177/0363546512453292

[115] European Medicines Agency. Hyalograft C autograft − Identification code EMA/25287/2013 2013.

[116] C. M. M. Peeters , M. J. C. Leijs , M. Reijman , G. J. V. M. van Osch , P. K. Bos , Osteoarthritis Cartilage 2013, 21, 1465. 23831631 10.1016/j.joca.2013.06.025

[117] M. M. Lalu , L. McIntyre , C. Pugliese , D. Fergusson , B. W. Winston , J. C. Marshall , J. Granton , D. J. Stewart , PLoS ONE 2012, 7, e47559. 23133515 10.1371/journal.pone.0047559 PMC3485008

[118] M. P. Prado , J. G. Kennedy , F. Raduan , C. Nery , Rev. Bras. Ortop. 2016, 51, 489. 27818968 10.1016/j.rboe.2016.08.007 PMC5091026

